# Process Mapping Midwifery Students’ Clinical Placement in Sierra Leone: Identifying Facilitators and Barriers

**DOI:** 10.5334/aogh.4441

**Published:** 2024-07-08

**Authors:** Julie Mann, Elizabeth Lemor, Frances Fornah, Patricia Juana-Kamara, Mary Augusta Fullah, Mustapha Sonnie, Brittney van de Water

**Affiliations:** 1 Seed Global Health, Boston, MA, USA; 2 Ministry of Health, Freetown, Sierra Leone; 3 School of Midwifery Makeni, Makeni, Sierra Leone; 4 School of Midwifery Bo, Bo, Sierra Leone; 5 Seed Global Health, Freetown, Sierra Leone; 6 Connell School of Nursing, Boston College, Chestnut Hill, MA, USA

**Keywords:** midwifery education, preceptors, clinical placement, clinical learning environment, sub-Saharan Africa, participatory action research, implementation science

## Abstract

*Background:* Improving midwifery education is critical to improving maternal and infant health outcomes in Sierra Leone. A significant priority within midwifery education is to strengthen the clinical teaching and students’ hands-on experience in the clinical setting.

*Objectives:* To identify facilitators and challenges within midwifery students’ clinical placements and to highlight areas to strengthen the clinical midwifery education system as well as the role of preceptors.

*Methods:* We conducted a participatory process mapping with two schools of midwifery in Sierra Leone to detail steps taken by practicing midwives and midwifery faculty when students are placed in clinical settings for midwifery rotations.

*Findings:* There were 42 participants from the Bo and Makeni regions of Sierra Leone. Participants included midwifery faculty from the Schools of Midwifery in Makeni and Bo, clinical midwives from two regional government hospitals, clinical midwives from two affiliated community health centers, and midwives from the District Health Management Teams. Three recurring themes emerged in the process. First, there was always some element of preparing or teaching the student. Second, there were administrative tasks to coordinate between the schools, clinical sites, and students, before, during, and after clinical placements. And third, there were elements of communication and collaboration between schools and clinical sites/preceptors that could be improved through shared understanding and standardization. Additional themes were inconsistencies across activities before, during, and after students’ clinical placement and limited opportunities and confusion around systems of evaluating all components of the clinical placement experience.

*Conclusions:* This study provides insight into the process of midwifery students’ clinical placement and highlights facilitators to be standardized and some modifiable barriers to be addressed. As Sierra Leone and many other similar countries in sub-Saharan Africa attempt to strengthen students’ clinical education through educating and developing preceptors, processing mapping can be a useful tool.

## Introduction

There is a global shortage of healthcare workers, with a projected shortfall of 10 million health workers by 2030 [1]. This shortage is especially seen in sub-Saharan Africa, where 25% of the global burden of disease lies, yet only 3% of the health workforce is based [2]. This shortage of healthcare workers is especially among nurses and midwives, who represent nearly 50% of the global health workforce and account for more than 50% of the current shortage of health workers [3, 4].

In order to meet Sustainable Development Goals 3.1 and 3.2 of reducing maternal and newborn mortality, this shortage needs to be urgently addressed. Educating and training midwives is the most effective way of addressing this critical shortage [4, 5].

Investment in midwifery education and training is a significant challenge. In 2019, the World Health Organization (WHO) published *Strengthening the Quality of Midwifery Education to Achieve Universal Health Care 2030* in which a framework was outlined to strategically guide investment [6]. A significant barrier in midwifery education identified in this report was that midwifery educators are more confident with theoretical classroom teaching than with clinical teaching [6]. The report also noted that midwifery schools and students lacked critical access to clinical settings, or simulation tools, to support competency-based education [6]. *The State of the World’s Midwifery Report (SOWM) 2021* found similar challenges in midwifery education [4]. This report identified a lack of investment in educators, limited skills and knowledge in contemporary teaching and learning, and inadequate hands-on experience for students as key barriers to midwifery education [4].

Preceptorship is one of the key strategies to foster midwifery education and training and gain more hands-on experience. Preceptorship is a process designed to pair students with instructors in the clinical setting. These instructors, called preceptors, are trained, experienced, and capable of supporting, educating, and strengthening student clinical training to ensure that skills needed are acquired during the training period [7]. Precepting has been embedded in midwifery education and training for decades [8]. According to the International Confederation of Midwives, midwifery students must spend at least 50% of their education in the clinical setting with preceptors [9].

Recognizing the importance of preceptorship in educating nurses and midwives, the Sierra Leone Nurses and Midwives Board, in collaboration with the Ministry of Health and Sanitation, designed policy and curricula to train nurses and midwives as preceptors in 2016. This was followed by the development of a training manual and a revised version of the 2016 curriculum in 2018. With this curriculum, the ministry has trained over 100 preceptors.

However, creating a curriculum and conducting training for preceptors is not enough. In Sierra Leone, effective preceptorship has been hampered by factors such as poor collaboration between hospitals and health training institutions, a lack of clinical instructors and preceptors for effective student supervision and clinical competency building, and weak frameworks for assessing students’ clinical performance [10]. Additional challenges have been heavy clinical workloads, leading to inadequate time in the workday for preceptors to teach students, and lack of motivation for midwives to become preceptors [11, 12].

Given that preceptorship is a critical component of midwifery education that goes beyond providing occasional preceptor training sessions, this study is designed to identify facilitators and challenges within the current midwifery student clinical placements in order to highlight areas to strengthen the role of preceptors and overall midwifery education. We aim to do this through process mapping the present processes of midwifery students’ clinical placement at two midwifery schools in Sierra Leone with midwifery stakeholders at each institution.

## Methods

### Study design

Process mapping and stakeholder engagement are important systems engineering techniques and implementation science methods used to identify steps during a defined process or sequence of operations (often clinical processes) and to bring stakeholders together and build consensus on the processes [13]. This is a prospective purposive study to assess current processes for student preceptorship training in the Schools of Midwifery in Bo and Makeni, Sierra Leone.

### Study sites and population

The location of the process mapping occurred over two days in February 2023 at the Bo and Makeni Schools of Midwifery. These sites were selected due to the forthcoming midwifery preceptor program which will be implemented at these sites, and the interest in mapping the current process for facilitating student clinical learning.

The study population was drawn from multiple facilities which will be implementing the forthcoming preceptor program, including staff from government hospitals, schools of midwifery, and affiliated community health centers.

### Data collection

Data was collected through facilitated discussions and active group work during two day-long retreats held at each midwifery school. Three main questions were posed to the group: (1) What is the process for facilitating students in the clinical setting before, during, and after their midwifery clinical rotation?; (2) What helps make the process of a student’s clinical rotation go smoothly?; and (3) What are the challenges for student midwifery clinical placements?

In both Bo and Makeni, participants were randomly divided into four small groups of four to six people. In total there were eight focus groups, four in Makeni and four in Bo. Groups discussed each question for approximately 10 to 15 minutes. Then, each small group elected a speaker to present to all participants their thoughts for each question. After groups presented, facilitators harmonized key points and garnered participant agreement through additional discussion.

### Analysis

Data was collated and organized into key themes. The program facilitators further discussed themes, combined repeated themes, and had follow-up meetings with the heads of the midwifery schools to clarify any unresolved points and to help with interpretation. The description of the process was delineated into concrete timeframes: “before,” “during,” and “after” clinical placement. The Standards for Quality Improvement Reporting Excellence (SQUIRE) guideline was used to guide the development of this research report.

### Ethics

Ethics approval was obtained in Sierra Leone (approved on February 1, 2023, by the Sierra Leone Ethics and Scientific Review Committee, Freetown, Sierra Leone) and the United States (approved on January 23, 2023, by Boston College in Boston; protocol number 23.147.01).

## Results

There were 42 participants: 20 from the Bo region and 22 from the Makeni region. Participants included midwifery faculty from the Schools of Midwifery in Makeni and Bo, clinical midwives from two regional government hospitals, clinical midwives from two affiliated community health centers, and midwives from the District Health Management Teams.

### Process for facilitating students in the clinical setting

In Tables 1–3, the current process for placement of students in the clinical setting is outlined into activities that midwifery schools, clinical sites, and/or students engage in before, during, and after clinical placements.

**Table 1:** Process for facilitating students before midwifery clinical placements.

BEFORE CLINICAL PLACEMENT
**Preparing Students**
Practical sessions held in skills labs. List of expected clinical competencies given to student. Review of expectations for student performance and behavior during clinical placement is given.
**Administrative Tasks**
School faculty assigns student clinical placement sites. School faculty writes letter with student roster and objectives for clinical site.
**Communication and Collaboration with Clinical Site and Preceptors**
Roster/objective letter is sometimes given to head of clinical site. Sometimes the head of clinical site shares roster/objective letter with preceptors. Sometimes schools call clinical sites to notify them of student roster/objectives. Placement of students in rural community health centers is often delayed.

**Table 2:** Process for facilitating students during midwifery clinical placements.

DURING CLINICAL PLACEMENT
**Teaching and Supervision of Students**
Students are sometimes oriented to clinical site. Preceptors teach, support, and supervise students when time allows ○ Types of teaching, support, and supervision: ▪ observation ▪ return demonstration ▪ asking questions to determine knowledge gaps ▪ review and discussion of student documentation in patient chart ▪ reflection/debriefing after clinic session Midwifery school faculty sometimes visit clinical site to observe and support students.
**Administrative Tasks**
Head nurse of the units sometimes informs preceptors of students’ clinical objectives. Preceptors are assigned to one or more students. Students are responsible for signing in and out of clinical to monitor daily attendance. Preceptors assign students to a day or night shift. Preceptors assign students to various locations in the clinic or hospital (e.g., postpartum unit, triage unit, operating theater). Preceptors sign off on student’s clinical proficiency in student log book.
**Communication and Collaboration with Clinical Site and Preceptors**
Students report to clinical placement site. Staff “compromise”/ make deals with students. Preceptors sometimes give feedback to school on student attendance and performance.

**Table 3:** Process for facilitating students after midwifery clinical placements.

AFTER CLINICAL PLACEMENT
**Student Issues**
Students return to school. Students prepare for exams.
**Administrative Tasks**
Preceptors review and sign students’ log books/portfolio books. Faculty review students’ log books/portfolio books.
**Communication and Collaboration with Clinical Site and Preceptors**
School faculty and preceptors occasionally meet to discuss a student’s performance. Students sometimes evaluate their clinical placement site. Students are sometimes asked to recommend good preceptors. Preceptors occasionally get feedback from students.

Students are assigned for clinical placement throughout the country. It is noted that expectations and objectives for students are reviewed prior to clinicals; however, this does not happen consistently across schools. Also, notification of students coming to clinical sites is sometimes done in writing and sometimes through a phone call, from the school to each site. Not all clinical sites receive a roster with names of students and their contact numbers. Delays in clinical placement of students assigned to rural community health centers might occur because the District Health Offices in each region are involved in placing these remote students. The addition of another person (e.g., the District Health Management Nurse, who assigns students to the remote clinics) in the process further delays, at times, the student’s assignment to a clinical site.

Clinical objectives are outlined in a “portfolio book.” The portfolio book is given to students before their first clinical placement and is where each student logs cases they experience throughout the clinical placement.

Preceptor teaching, support, and supervision is noted to occur when time allows, but participants stated preceptors are often too busy with patient care or administrative tasks. Preceptors use a variety of methods to teach and support students when time allows. During the clinical rotation, ideally one preceptor is assigned to approximately five students, but often a preceptor has more than five students due to many schools sending students to one clinical site. Often supervision from midwifery school faculty does not occur because of time constraints and logistical issues such as lack of fuel to travel to clinical sites.

Hospital- and clinic-based preceptors are also responsible for assigning students to locations within the clinic where they can achieve different clinical objectives. These student assignments are called duty rosters. Students may be placed in the maternity ward to assist with deliveries, the operating theater, the postpartum unit, or anywhere else that the preceptor sees fit. When assignments are made, it is noted by participants that sometimes students do not adhere to their assignments.

In terms of communication and collaboration during clinical placement, students are expected to report to their site each day; however, participants stated that sometimes students fail to report and never inform the school or their preceptor of their absence. One school has a system where students Whatsapp their school to verify daily attendance or absences.

At times during clinical rotations, preceptors “compromise” or make deals with students. For example, students might have a familial relationship to a preceptor or an important member of the community, so the preceptor might give this student special treatment and not report the student’s absence or poor/inappropriate performance to the school.

After clinical placements, preceptors are supposed to review all student clinical cases logged in their portfolio books. In the same books, they are also supposed to sign off on whether a student is clinically competent in specific areas. Participants noted that sometimes preceptors fail to sign off students in these log books and at times, students sign the log books themselves, verifying their own competency. Schools and preceptors informally and occasionally communicate directly about students’ performances or the clinical rotation experience. Participants again noted that “compromises” around honest feedback of students’ performances occur. Sometimes preceptors inflate students’ performances for money or because the student is a relative or of high importance in the community.

### Facilitators and challenges in midwifery clinical rotations

#### Ideal facilitators in the process of a student’s clinical rotation

In Table 4, participants identified facilitators that, if present, enable a smooth process of clinical placement for midwifery students. As noted in Tables 1 through 3, some of these facilitators already exist in the current clinical placement process.

**Table 4:** Ideal facilitators of a midwifery student’s clinical placement process.

Adequate number of preceptors for midwifery students A well-defined, standardized, national preceptorship program Clear roles and responsibilities of a preceptor Memorandum of understanding between midwifery schools and clinical sites Standardized communication system between clinical facilities, preceptors, students, and schools Timely update of policies and guidelines are communicated to preceptors Students come to clinicals with their own equipment and consumables Preceptors give training in educational theory Schools and clinical sites hold students accountable for their actions Having housing arrangements for students in rural community health centers Standardized system for all students when they report to clinical placement Preceptors have access to the school simulation lab to teach students Office space for preceptors at clinical site Supportive culture for preceptors *National Midwifery Procedure Manual* to standardize midwifery education Adequate time in clinical placement for students to meet learning objectives Code of conduct for students and preceptors

**Figure 1:**
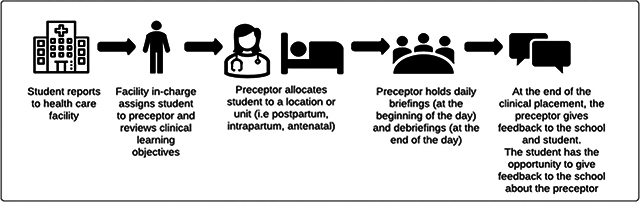
Ideal process for a student’s clinical placement.

Participants discussed that a standardized system for all students when they report to clinical placement would be helpful. They suggested this process could look something like Figure 1.

Participants also desired a more supportive culture for preceptors. They wanted preceptors to be recognized as a respected and desirable career pathway in midwifery. They suggested that preceptors should be promoted at similar levels as faculty/other educators and suggested preceptors should be given compensation for precepting: money, consumables, time, and/or recognition by the government, school, or clinical facility. And they should be provided with refresher trainings (i.e., continuing professional development). They desired to be connected with other preceptors in the country and region through preceptor conferences and professional opportunities. They desired a more supportive culture in the clinical setting, where preceptors could teach one another how to be better educators. One participant stated that she wished there would be no jealousy among preceptors and went on to say, “If you think a preceptor is doing a good job, get closer to her.”

Participants desired a designated office for a multipurpose precepting space at clinical sites to hold teaching sessions, provide student feedback, and to facilitate daily reflection/debriefing sessions. They also desired clinical placements to be longer, as it is challenging to achieve clinical objectives in the current timeframe.

#### Existing challenges for student midwifery clinical placements

After mapping the existing process for midwifery student clinical placement and identifying ideal facilitators of that process, participants shared perceived challenges in the current process. These challenges are listed in Table 5.

**Table 5:** Current challenges identified in the midwifery student clinical placement process.

Lack of clear communication between students, schools, preceptors, and clinical sites Clinical rotations are too short to achieve clinical objectives Limited physical space to accommodate students at clinical sites Large numbers of students sent to the same clinical sites Inadequate teaching, support, and supervision of students by preceptors Inadequate teaching, support, and supervision of students by midwifery faculty Lack of transportation for students to clinical sites No housing for students in rural clinical sites Unmotivated students Unmotivated preceptors Preceptors “compromise” with students in exchange for benefits or money Preceptors not being up-to-date in clinical skills and evidenced-based protocols Students and preceptors not following clinical objectives outlined by school Students are not evaluated according to required competencies Limited equipment, medications, and supplies (e.g., blood pressure cuffs, gloves) Students are not punctual in arriving at clinical sites each day Preceptors are not punctual in arriving at clinical sites each day Preceptors lack ownership of preceptor assessment Preceptors bullying students

A major challenge is the current lack of clear communication between students, schools, preceptors, and clinical sites. Participants described that there is no clear and consistent system in which preceptors can notify schools of students’ absences or performance even when memorandums of understanding are in place. Clinical sites are not always informed of students’ date of arrival, number of students, or names of students. Daily student assignments (i.e., “duty rosters”) are not made or communicated to students, which participants felt should be the responsibility of preceptors. There are limited formalized opportunities for schools and students to give feedback to preceptors regarding their precepting skills and style. Sometimes there are no memorandums of understanding between the schools and clinical sites outlining expectations—this occurs more often at private healthcare facilities and rural health clinics.

Another major challenge is that there is inadequate teaching, support, and supervision of students by preceptors due to many various reasons. Some reasons given were there are too many students, preceptors are too busy with patient care, and that there are not enough preceptors. Additionally, it was noted that preceptors have limited knowledge of how to teach in the clinical setting. There is also the challenge of inadequate teaching, support, and supervision of students by midwifery school faculty due to time constraints, distance from school to some clinical sites, and competing demands of faculty with high workloads.

Unmotivated preceptors are another identified challenge. Participants stated this lack of motivation can stem from lack of compensation for acting as a preceptor, lack of respect that students give preceptors, or preceptors not wanting to precept but being told they must by ward matrons or schools. It was noted that preceptors get very limited support from the midwifery schools and clinical sites.

Also, there is a general lack of equipment and supplies at clinical sites, making it difficult to involve students in patient care at times or to demonstrate and teach best practices. And as one participant stated, students come and often “waste” valuable clinical supplies.

Preceptors vocalized that they do not feel they have authority from midwifery schools to assess and communicate student competencies on behalf of midwifery schools. For example, sometimes preceptors’ evaluations indicate only whether a student has or has not performed a skill or procedure as opposed to grading or evaluating the student on their level of performance.

As previously mentioned, another challenge is that preceptors sometimes compromise or make deals with students, such as inflating grades or not notifying schools of student absences in exchange for money or other benefits.

## Discussion

This study provides insight into the process of midwifery students’ clinical placement and highlights facilitators to be standardized and some modifiable barriers to be addressed, all of which were identified by midwifery stakeholders in Sierra Leone.

Process mapping is a useful tool used in implementation science for participants to gather a shared understanding of the reality of a situation, identify improvement opportunities, engage stakeholders in an issue, define objectives, and increase learning and empathy of stakeholders [14]. Three recurring themes emerged in this process-mapping activity. First, there was always some element of preparing or teaching the student. Second, there were administrative tasks to coordinate between the schools, clinical sites, and students, before, during, and after clinical placements. And third, there were elements of communication and collaboration between schools and clinical sites/preceptors that could be improved through shared understanding and standardization.

The theme of clear communication and collaboration between the midwifery school, preceptors at clinical sites, and students stood out as one of the most challenging and critical areas in the student clinical placement process. Communication was highlighted as a facilitator if it were clear, well-defined, and standardized; yet communication was considered a barrier if not. This study is not alone in identifying communication and collaboration as important yet challenging in midwifery education [15–18]. Providing opportunities for school faculty and clinical preceptors to discuss preferred methods of communication (e.g., formal letters, email, WhatsApp, phone calls) and who the communication channels should go through (e.g., school leadership to clinical site leadership or course lecturer to clinical placement ward/preceptor) is important to include in memorandums of understanding. Given that these documents already exist, adapting and updating them with more specifics is an actionable and modifiable next step.

Throughout process mapping, inconsistencies were noted across activities before, during, and after students’ clinical placement. Participants frequently noted that certain identified activities “sometimes” or “occasionally” occur or do not occur. For example, *sometimes* students were oriented to clinical sites; *sometimes* clinical sites received a student’s name and contact information; *sometimes* midwifery school faculty visited a clinical site to observe and support a student. When such inconsistencies exist, students are confused about expectations and whom to report to or receive guidance from. Also, preceptors are unsure about their roles. A systematic review of clinical learning environments in nursing and midwifery confirmed that such lack of consistencies, especially in the area of planning and organizing clinical placements, is a significant barrier to creating a positive learning environment [19]. Creating standard operating procedures for the clinical placement process similar to Figure 1 above, where participants in this study outlined the ideal process for student clinical placement, could be beneficial and address this challenge. Additionally, inconsistencies in clinical expectations for students could be addressed with ensuring midwifery curricula are standardized according to the International Confederation of Midwives (ICM) Essential Competencies for Midwifery Practice and ICM’s Global Standards for Midwifery Education [9, 20].

Part of the inconsistencies noted was that there are limited opportunities and confusion around systems of evaluating all components of the clinical placement experience. Participants reported that preceptors often do not have the opportunity—nor authority—to evaluate the students they have the primary responsibility for precepting. This incongruence in tasks can cause confusion and has been cited as demotivating [21–23]. In business and project management the RACI matrix, which stands for responsible, accountable, consulted, informed, is a well-known tool to ensure clear communication and smooth workflows across all parts of a team [24]. Although there is no evidence in the literature yet, such a tool as the RACI matrix could be useful in the context of the clinical education placement process to clarify roles and responsibilities.

As a recent study in Sweden found, good preceptorship occurs when midwives achieve full self-efficacy, when they master the preceptor role, and when they have the ability to help the student reach confidence and clinical independence [25]. In order for this to occur, preceptors must be given autonomy to fulfill their duties as preceptor and be given the responsibility to assess, in a meaningful way, student performance, thus providing motivation for preceptors, schools, and students to recognize preceptors with a clear role and responsibilities. After these are defined and established, there is potential for a decreased burden on school lecturers’ time and school cost for transportation and per diem rates. This could have the potential to start incentivizing and paying preceptors for their time or supplementing some of the time in the clinical setting they are spending away from direct patient care and teaching, precepting, and debriefing with students, and if not paying preceptors directly, perhaps providing necessary equipment or continuing professional development opportunities [26, 27].

### Limitations

One limitation of this study is that no student perspective is captured in this process mapping, and they are important stakeholders in this process. Additionally, representatives from only two midwifery institutions and clinical sites from 2 of the 16 regions of Sierra Leone participated in the study, limiting generalizability of findings. This participatory method was also conducted during the launch of the preceptor program; therefore, stakeholders may hold some bias toward precepting and may have over- or understated some facilitators or challenges.

## Conclusions

Despite numerous studies stressing the importance of training nursing and midwifery preceptors in order to strengthen nursing and midwifery education, it is not the sole determining factor in students’ success and achievement of competency [28]. The clinical learning process is complex. As noted in a study that looked at why a nursing preceptor program failed in South Africa, they stressed the importance of a systems approach to strengthening clinical learning and the importance of examining the context within which preceptors are expected to support and educate students [28].

As Sierra Leone and many other similar countries in sub-Saharan Africa attempt to strengthen students’ clinical education through educating and developing preceptors—the core educators in the clinical setting—mapping the clinical placement process and identifying facilitators and barriers in that process is essential.
